# Acute and Chronic Workload Ratios of Perceived Exertion, Global Positioning System, and Running-Based Variables Between Starters and Non-starters: A Male Professional Team Study

**DOI:** 10.3389/fpsyg.2022.860888

**Published:** 2022-03-17

**Authors:** Hadi Nobari, Nader Alijanpour, Alexandre Duarte Martins, Rafael Oliveira

**Affiliations:** ^1^Department of Physiology, School of Sport Sciences, University of Extremadura, Cáceres, Spain; ^2^Department of Exercise Physiology, Faculty of Educational Sciences and Psychology, University of Mohaghegh Ardabili, Ardabil, Iran; ^3^Sports Scientist, Sepahan Football Club, Isfahan, Iran; ^4^Sports Science School of Rio Maior, Polytechnic Institute of Santarém, Rio Maior, Portugal; ^5^Life Quality Research Centre, Rio Maior, Portugal; ^6^Departamento de Desporto e Saúde, Escola de Saúde e Desenvolvimento Humano, Comprehensive Health Research Centre (CHRC), Universidade de Évora, Évora, Portugal; ^7^Research Centre in Sport Sciences, Health Sciences and Human Development, Vila Real, Portugal

**Keywords:** ACWR, EWMA, coupled, uncoupled, GPS, high speed, RPE, player status

## Abstract

The study aim was 2-fold (i) to describe and compare the in-season variations of acute: chronic workload ratio (ACWR) coupled, ACWR uncoupled, and exponentially weighted moving average (EWMA) through session-rated perceived exertion (s-RPE), total distance (TD), high-speed running distance (HSRD), and sprint distance across different periods of a professional soccer season (early, mid, and end-season) between starters and non-starters; (ii) to analyze the relationship the aforementioned measures across different periods of the season for starters and non-starters. Twenty elite soccer players (mean ± SD age, 29.4 ± 4.4 y; height, 1.8 ± 0.1 m; and body mass, 74.8 ± 2.3 kg). They were divided into starter and non-starter groups and were evaluated for 20 weeks. ACWR had general changes throughout the season. At the beginning and end of the mid-season, the highest ACWR was observed in three parameters: s-RPE, TD, and HSRD. ACWR and EWMA through sprint distance were higher at the beginning of the early-season than at any other time of the season.

The ACWR coupled of s-RPE shows a significant higher value for non-starters than starters (*p* = 0.015; *g* = −1.01 [−1.98, −0.09]) and the ACWR coupled of TD shows a significant higher value for starters than non-starters in early-season (*p* < 0.01; *g* = 3.01 [1.78, 4.46]) and shows a significant higher value for non-starters than starters in mid-season (*p* < 0.01; *g* = −2.52 [−3.83, −1.39]), and end-season (*p* < 0.01; *g* = −2.57 [−3.89, −1.43]). While the EWMA of TD shows a significant higher value for starters than non-starters in early-season (*p* < 0.01; *g* = 2.25 [1.17, 3.49]) and mid-season (*p* < 0.01; *g* = 2.42 [1.31, 3.71]), and shows a significant higher value for non-starters than starters in end-season (*p* < 0.01; *g* = −2.23 [−3.47, −1.16]). Additionally, we found some correlations between external and internal load measures during three periods of the in-season. The study’s main finding was that the indexes of ACWR and EWMA were useful to detect differences between period and between playing status with the exception for the sprint variable. In addition, the necessary work for non-starter players’ improvement is not done during training, and these players lose their readiness as the season progresses. Consequently, these players perform poorly during the match. Therefore, coaches and their staff should consider devising new activities to keep non-starter players physically fit. This deficit must be accounted for in training because they compete in fewer matches and have less burden than starters.

## Introduction

Nowadays, monitoring and assessment of professional soccer players are common and mandatory practices to quantify the impact of training and match loads on the players (Miguel et al., 2021) and for better load adjustments (Burgess, 2017; West et al., 2020).

The quantification of external and internal load allows to determine intra- and inter-week variations of the players (Akenhead and Nassis, 2016). Some examples of external load could be associated with the global positioning system (GPS) measures such total distance and running distance variables while internal load is associated with the external load effect of the body that could be measure by rated perceived exertion (RPE) or hear rate (Miguel et al., 2021).

One way to identify intra- and inter-week variations is through the acute: chronic workload ratio (ACWR) that provided the relationship between the load of the last/current week (acute load) with the load of the last 4 weeks (28 days, chronic load). By other words, this version of ACWR uses a coupled formula, which consist in dividing the acute workload (i.e., the 1-week rolling workload data), by the chronic workload (i.e., the rolling 4-week average workload data; Gabbett et al., 2016; Hulin et al., 2016). Another way to identify week variations is through the uncoupled version of ACWR. In this version, chronic load does not consider the most recent week which means that weekly acute workload (i.e., the accumulated daily loads during 1 week) is divided by the weekly chronic load (i.e., average of the three preceding weeks; Windt and Gabbett, 2019). Finally, the most recent way to analyze such variations is through the exponentially weighted moving average (EWMA; Williams et al., 2017). EWMA also contemplated the calculation of acute and chronic loads, but it attributes a decreasing weight for older load values. This detail accounts for loss of fitness and gain of fatigue over time (Williams et al., 2017).

Originally, the developments of such ratios were based to predict injury risk; however, recent research found limitations in such predictions (Fanchini et al., 2018; Impellizzeri et al., 2020). Based on that, some longitudinal studies in soccer have been reporting seasonal variations of those workload measures (Nobari et al., 2021a,e,g; Oliveira et al., 2021b). But those studies had some similar limitations such the small sample size or the one team analysis which makes difficult to generalize results and it suggests that more studies should be developed in this field.

Nonetheless, the studies mentioned before found several variations over the season. For instance, two studies analyzed ACWR of player load measure for starters and non-starters throughout the early-, mid-, and end-season periods of a professional team. The authors found significant differences between season periods for ACWR with higher values being found at the beginning of the season. Starters showed little variation while non-starters displayed higher variation across the season (Nobari et al., 2021a,e). Other study conducted in under-16 players also analyzed ACWR of session-RPE (s-RPE) through early-, mid-, and end-season periods and found higher values in early > mid > end-season (Nobari et al., 2021g). With a different approach, a study in under-17 soccer players found similar values during 10 mesocycles of the in-season (Martins et al., 2021).

As mentioned before, those ratios could be influenced by several situational factors such as players status (starter or non-starter players). For example, two studies (Nobari et al., 2021a,e) found higher values for starters than non-starters in early-season period through the ACWR calculated with body load, while Oliveira et al. (2021b) did not any significant difference between player status across 10 mesocycles of the in-season through the ACWR calculated through s-RPE, total distance (TD), and high-speed running distance (HSRD). Moreover, in under-17 soccer players, it was only found two differences between starters and non-starters across 10 mesocycles of the in-season for ACWR of s-RPE. Specifically, one mesocycle showed higher values for starters and another for non-starters (Martins et al., 2021).

Furthermore, the analysis of playing status emerged through other variables such as monotony, strain, or accumulated load. Even so, it has been showed a tendency of higher values for starters compared to non-starters (Nobari et al., 2020, 2021d,f).

To the best knowledge of the authors, only one study analyze ACWR ratios through the measures of s-RPE (internal load), HSRD, and total distance (TD) but without the use of uncoupled ACWR or EWMA calculations (Oliveira et al., 2021b). For those reasons, it is necessary to produce research that analyze those ratios and that tries finding out the relationships between external and internal measures.

Therefore, the purpose of this study was 2-fold: (a) to describe and compare the in-season variations of ACWR coupled, ACWR uncoupled, and EWMA through s-RPE, TD, HSRD, and sprint distance across different periods of a professional soccer season (early-season, mid-season, and end-season) between starters and non-starters; (b) to analyze the relationship the aforementioned measures across different periods of the season for starters and non-starters. We hypothesized that the weekly workload variations in starters would be greater than in non-starters and that starters would withstand more acute and chronic loads than non-starters in all periods of the season. Additionally, we hypothesized that internal workload ratios would be correlated with external workload ratios.

## Materials and Methods

### Participants

Twenty elite soccer players from the First League of Iran (Asian) participated in this study. They were divided into two groups: starters (*n* = 10, age 30.0 ± 4.9 years, 1.80 ± 0.02 m, and 73.6 ± 1.5 kg) and non-starters (*n* = 10, age 28.8 ± 3.9 years, 1.8 ± 0.1 m, and 76.4 ± 3.3 kg). The inclusion criteria were regular participation in 80% of weekly training sessions (Clemente et al., 2017). The exclusion criteria included as: (i) players with prolonged injury or a lack of participation in training for at least two consecutive weeks (two players were removed based on this criterion); (ii) goalkeepers were excluded from the study due to differences in training and match demands (one player was removed bases on this criteria).

The criteria to define starters and non-starters were assessed week by week to a player’s attendance time in three consecutive matches (≥60 min in each match), while non-starters were considered those who did not achieve this duration based on previous studies (Martins et al., 2021; Nobari et al., 2020, 2021c; Oliveira et al., 2021a,b).

All participants were familiarized with the training protocols prior to investigation. Moreover, they provided written consent to participate in this study which was conducted according to the requirements of the Declaration of Helsinki and was approved by the University of Isfahan research ethics committee.

### Experimental Design

The present study is a cohort study with a descriptive-longitudinal approach. The players were monitored for 20 consecutive weeks during in-season. For the purposes of the present study, all training sessions conducted during the main team sessions were considered. Data from rehabilitation or recuperation were excluded. Duration of training sessions included warm-up stages (jogging, stretching in the large leg and upper body muscles, working with the ball under the supervision of technical staff for warm-up, and dynamic stretching, 10–15 m sprints), the main stage of training (included tactic exercises under the supervision of the head coach and technical staff or physical exercises in the weight room or on the training ground under the supervision of the head coach and technical staff), and cooling down (jogging, static stretching, muscle relaxation, and trying to reduce heart rate). Researchers standardized only the first and last 30 min (before and after each training session). Information on all stages of the exercise was recorded using a GPS device and transferred to a computer for review.

This study includes data from the beginning of the early-season (30 October 2017) that lasted until the end of the season (18 March 2018). The in-season was organized into three periods: early-season (weeks 1–7); mid-season (weeks 8–13); and end-season (weeks 14–20; Table 1).

**Table 1:** Description of the present study.

Phases of the season	Early-season	Mid-season	End-season
Number of weeks	7	7	6
Training sessions (n)	15	14	18
Training duration, average minutes, ST	62.13	73.56	79.55
Training duration, average minutes, NST	61.58	73.25	77.40
Training duration, total minutes, ST	1031.60	1271.60	1900.70
Training duration, total minutes, NST	1004.80	1281.70	1903.00
Number of matches (N)	7	8	5

ST, Starters; NST, Non-starters.

The number of the weeks and training sessions, number of competitive matches, and total training duration (in average and total values) for starters and non-starters are presented in Table 1.

### External Load Monitoring

During the season, all training and match sessions were monitored using GPS [GPSPORTS systems Pty Ltd., Model: SPI High-Performance Unit (HPU); Australian]. This model includes 15 Hz position GPS and a tri-axial accelerometer. According to a previous study, this device has a high validity and reliability (Williams and Tessaro, 2018). There were no reported adverse weather conditions to affect data collection. Prior to the start of the match, belts were placed on the players’ shoulder and chest. After each cool down session at the end of the training, the belts were collected from the players. All belts were checked by the team’s GPS manager and then entered into the dock system to download the information, which was then stored on the computer with the Team AMS software. The data from each session were automatically deleted from the belt memory after download. Prior to the next session, the belts were placed in an electric charge station. The SPI IQ Absolutes were adjusted for GPS default zone throughout the season. Also, the personal characteristics (such as height and weight) of each player were entered in the software and each player registered a belt in his own name for using until the end of the season.

### Internal Load Monitoring

Players were daily monitored for their RPE using the CR-10 Borg’s scale (Borg, 1970), adapted by Foster et al. (2001). Previous study demonstrated the validity and reliability of this scale to estimate the session intensity (Kelly et al., 2020). Thirty minutes after the end of each training session, players rated their RPE value using an app on a tablet. The scores provided by the players were also multiplied by the training duration to obtain the s-RPE (Bresnan and Mchombo, 1995; Foster et al., 2001). The players were previously familiarized with the scale, and all the answers were provided individually to avoid non-valid scores.

### Calculations of Training Indexes

Through s-RPE, total distance, HSRD, and sprint distance, the following variables were calculated as: (i) ACWR, using coupled formula: dividing the acute workload (i.e., the 1-week rolling workload data), by the chronic workload (i.e., the rolling 4-week average workload data; Hopkins et al., 2009; Dupont et al., 2010; Impellizzeri et al., 2020; Myers et al., 2020; Dalen-Lorentsen et al., 2021); (ii) ACWR using uncoupled formula: dividing the weekly acute workload (i.e., the accumulated daily loads during 1 week), by the weekly chronic load (i.e., average of the three preceding weeks; Windt and Gabbett, 2019); and (iii) exponentially weighted moving averages (EWMA; Williams et al., 2017). The EWMA for a given day was calculated as:


$$EWMA_{\mathrm{today}}=Load_{\mathrm{today}}\times \lambda_{a}+\left(\left(1-\lambda_{a}\right)\times EWMA_{\mathrm{yesterday}}\right)$$

Where $\lambda_{a}$ is a value between 0 and 1 that represents the degree of decay, with higher values discounting older observations in the model at a faster rate. The $\lambda_{a}$ is calculated as:


$$\lambda_{a}=2∕\left(N+1\right)$$

Where N is the chosen time decay constant, typically 7 and 28 days for acute (“fatigue”) and chronic (“fitness”) loads, respectively (Murray et al., 2017; Williams et al., 2017).

### Statistical Analysis

Descriptive statistics were used as mean ± standard deviation (SD) and 95% confidence interval (CI) to characterize the sample. Shapiro–Wilk was used to test normality of results. The relationship between all variables at the different periods was verified using bivariate correlations through Pearson product–moment correlation coefficient (*r*; Cohen and Cohen, 1983). The effect size of the correlations was determined by the following thresholds: <0.1 = trivial; 0.1–0.3 = small; > 0.3–0.5 = moderate; > 0.5–0.7 = large; > 0.7–0.9 = very large; and > 0.9 = nearly perfect (Batterham and Hopkins, 2006; Hopkins et al., 2009).

All variables obtained a normal distribution (Shapiro–Wilk>0.05). For that reason, repeated measures ANOVA followed by Bonferroni post-hoc test were used to compare variables for periods of the in-season and groups [2 groups (starters vs. non-starters) × 3 data points (early vs. mid vs. end-season)] (Nobari et al., 2020; Martins et al., 2021; Oliveira et al., 2021b; Nobari et al., 2021c). The results were significant for a *p* ≤ 0.05. Hedge’s g effect size (ES) was also calculated to determine the magnitude of pairwise comparisons. The following criteria was used as: The Hopkins threshold was utilized as follows: *g* ≤ 0.2, trivial; 0.2 *< g* ≤ 0.6, small; 0.6 *< g* ≤ 1.2, moderate; 1.2 *< g* ≤ 2.0, large; 2.0 *< g* ≤ 4.0, very large; and *g >* 4.0, nearly perfect (Hopkins et al., 2009).

All data were analyzed using IBM SPSS Statistics [version 22, IBM Corporation (SPSS Inc., Chicago, IL)].

## Results

**Figure 1:**
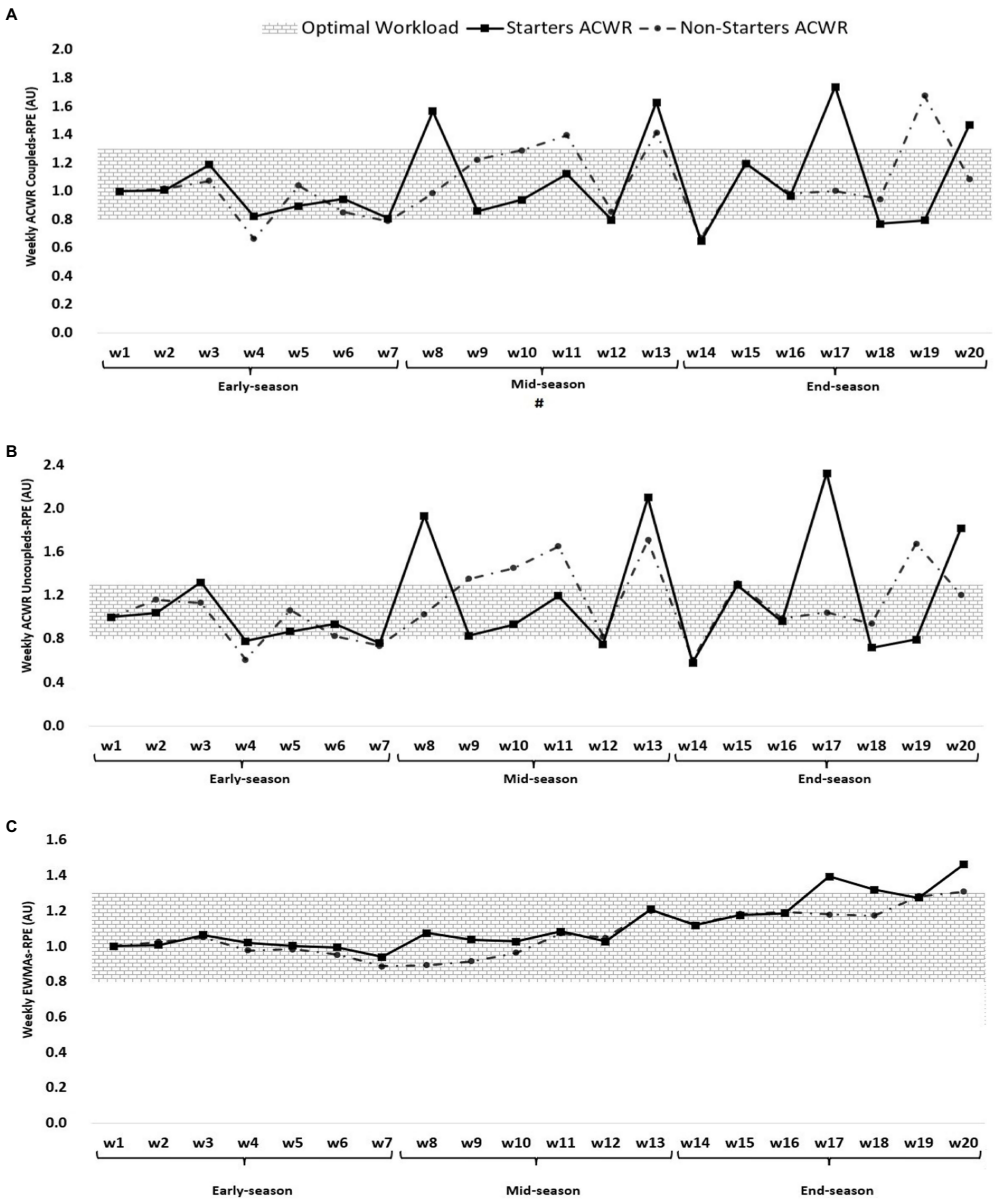
ACWR coupled **(A)** and uncoupled **(B)**, and EWMA **(C)** variations calculated through the s-RPE across 20-week starters and non-starters. ^#^Denotes significant difference between starters and non-starters.

**Figure 2:**
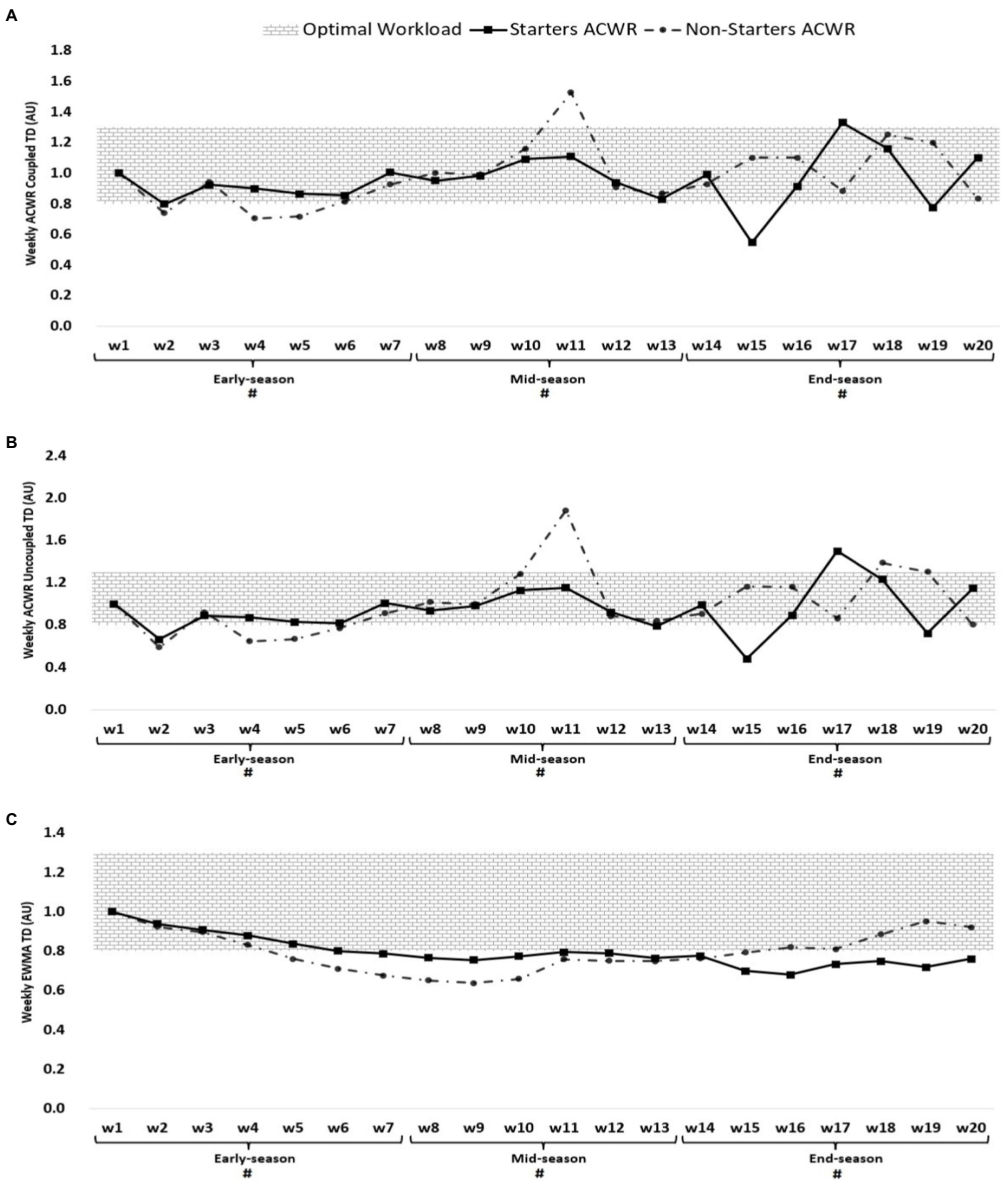
ACWR coupled **(A)** and uncoupled **(B)**, and EWMA **(C)** variations calculated through the total distance across 20-week starters and non-starters. ^#^Denotes significant differences between starters and non-starters.

**Figure 3:**
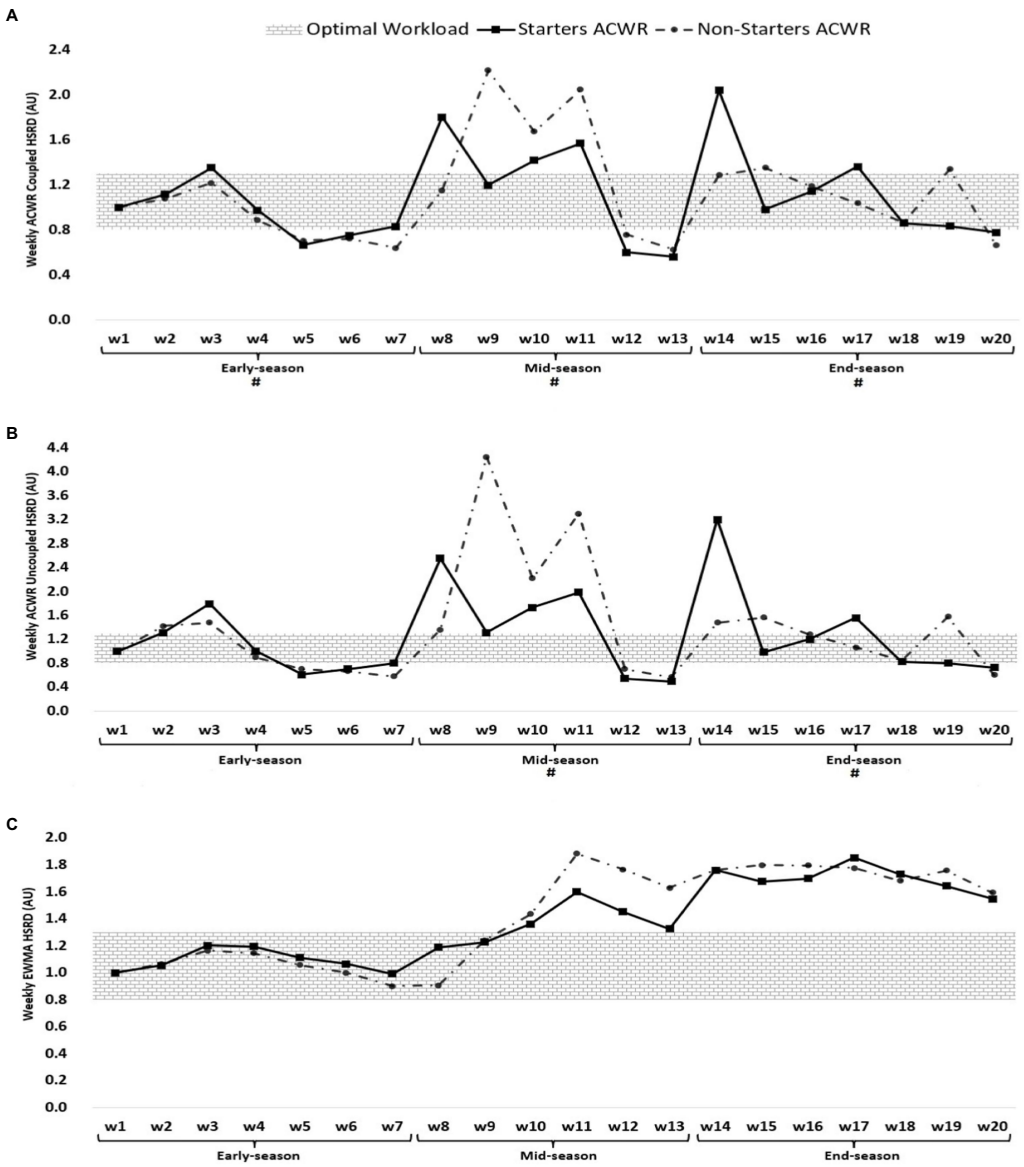
ACWR coupled **(A)** and uncoupled **(B)**, and EWMA **(C)** variations calculated through the HSRD across 20-week starters and non-starters. ^#^Denotes significant differences between starters and non-starters.

**Figure 4:**
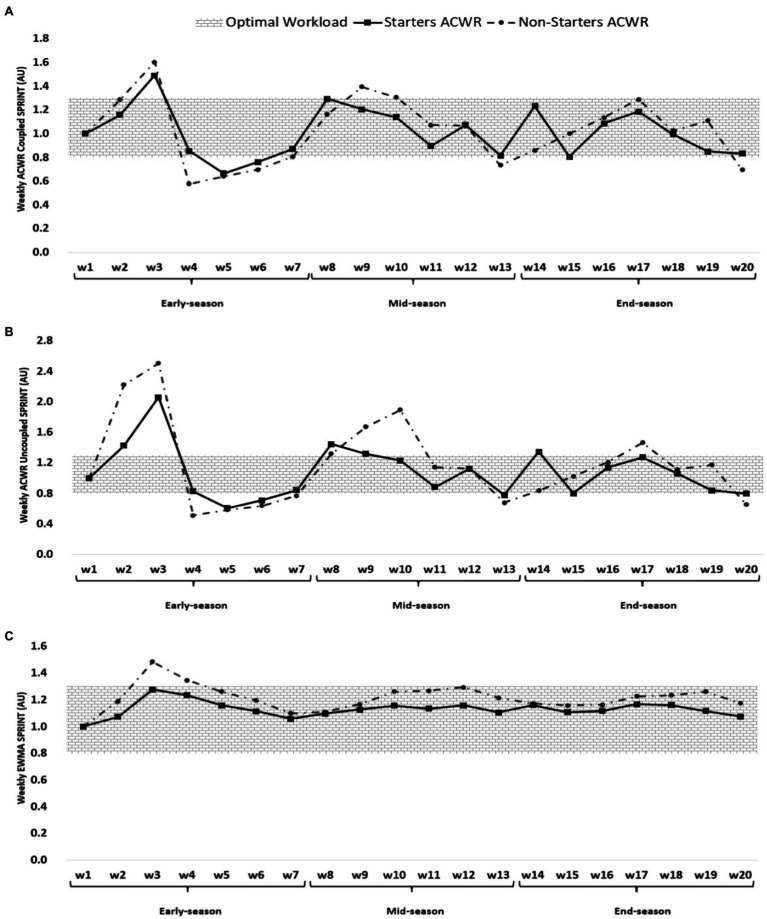
ACWR coupled **(A)** and uncoupled **(B)**, and EWMA **(C)** variations calculated through the sprint distance across 20-week starters and non-starters.

Figures 1–4 show an overall view of the weekly average for ACWR coupled, ACWR uncoupled, and EWMA calculated through s-RPE, total distance, HSRD, and sprint distance across different periods of a professional soccer season (early-season, mid-season, and end-season). Overall, Figure 1 shows that the highest ACWR coupled of s-RPE occurred in week 17 (1.74 AU, end-season) and week 19 for non-starters (1.68 AU, end-season), while the lowest value occurred in week 14 for starters (0.67 AU, end-season) and week 4 for non-starters (0.66 AU, early-season). The highest ACWR uncoupled of s-RPE occurred in week 17 (2.32 AU, end-season) for starters and week 13 (1.71 AU, mid-season), while the lowest value occurred in week 14 (0.58 AU, end-season) for starters and weeks 4 and 14 (0.61 AU, early and end-season, respectively). The highest EWMA of s-RPE occurred in week 20 (end-season) for both starters and non-starters (1.46 and 1.31 AU, respectively), while the lowest value occurred in week 7 (early-season) for both starters and non-starters (0.94 and 0.89 AU, respectively).

Figure 2 shows that the highest ACWR coupled of total distance occurred in week 17 (1.33 AU, end-season) and week 11 for non-starters (1.53 AU, mid-season), while the lowest value occurred in week 15 for starters (0.55 AU, end-season) and week 4 for non-starters (0.70 AU, early-season). The highest ACWR uncoupled of TD occurred in week 17 (1.50 AU, end-season) for starters and week 11 (1.88 AU, mid-season), while the lowest value occurred in week 15 (0.48 AU, end-season) for starters and weeks 2 for non-starters (0.59 AU, early-season). The highest EWMA of TD occurred in week 1 (early-season) for both starters and non-starters (1.00 AU), while the lowest value occurred in week 16 for starters (0.68 AU, end-season) and week 9 for non-starters (0.69 AU, mid-season).

Figure 3 shows that the highest ACWR coupled of HSRD occurred in week 14 (2.04 AU, end-season) and week 9 for non-starters (2.22 AU, mid-season), while the lowest value occurred in week 13 (mid-season) for both starters and non-starters (0.56 and 0.63 AU, respectively). The highest ACWR uncoupled of HSRD occurred in week 14 (3.19 AU, end-season) for starters and week 9 (4.24 AU, mid-season), while the lowest value occurred in week 7 (early-season) for both starters and non-starters (0.49 and 0.56 AU, respectively). The highest EWMA of HSRD occurred in week 17 (1.85 AU, end-season) and week 11 for non-starters (1.88 AU, mid-season), while the lowest value occurred in week 7 (early-season) for both starters and non-starters (0.99 and 0.90 AU, respectively).

Figure 4 shows that the highest ACWR coupled of SD occurred in week 3 (early-season) for both starters and non-starters (1.49 and 1.60 AU, respectively), while the lowest value occurred in week 5 for starters (0.67 AU, early-season) and week 4 for non-starters (0.58 AU, early-season). The highest ACWR uncoupled of sprint distance occurred in week 3 (early-season) for both starters and non-starters (2.06 and 2.51 AU, respectively), while the lowest value occurred in week 5 (0.61 AU, early-season) for starters and week 4 for non-starters (0.51 AU, early-season). The highest EWMA of sprint distance occurred in week 3 (early-season) for both starters and non-starters (1.28 and 1.48 AU, respectively), while the lowest value occurred in week 1 (early-season) for both starters and non-starters (1.00 AU).

Table 2 shows differences between starters and non-starters during the periods of the in-season for all variables. Regarding ACWR uncoupled of s-RPE, EWMA of s-RPE, EWMA of HSRD, ACWR coupled of sprint distance, ACWR uncoupled of sprint distance, and EWMA sprint distance, there were no significant differences between starters and non-starters.

**Table 2:** Differences between starters and non-starters during the periods of the in-season, mean ± SD [95% CI].

Measures	Early-season	Mid-season	End-season
ACWR CP s-RPE (AU), ST	0.95 ± 0.04 [0.92; 0.99]	1.15 ± 0.02 [1.13; 1.18]^*^	0.95 ± 0.05 [0.91;0.99]
ACWR CP s-RPE (AU), NST	0.92 ± 0.06 [0.89; 0.95]	**1.19 ± 0.05 [1.17; 1.22]**	0.91 ± 0.08 [0.87; 0.95]
ACWR UCP s-RPE (AU), ST	0.96 ± 0.06 [0.89; 1.02]	1.29 ± 0.04 [1.25; 1.33]	1.21 ± 0.08 [1.14; 1.28]
ACWR UCP s-RPE (AU), NST	0.93 ± 0.12 [0.87; 0.99]	1.33 ± 0.08 [1.29; 1.37]	1.11 ± 0.13 [1.04; 1.19]
EWMA s-RPE (AU), ST	1.00 ± 0.07 [0.94; 1.07]	1.08 ± 0.11 [0.99; 1.17]	1.28 ± 0.06 [1.22; 1.33]
EWMA s-RPE (AU), NST	0.98 ± 0.12 [0.92; 1.05]	1.02 ± 0.15 [1.15;1.27]	1.21 ± 0.11 [1.15; 1.27]
ACWR CP TD (AU), ST	**0.91 ± 0.02 [0.89; 0.92]**^§^	**0.98 ± 0.02 [0.96; 1.01]**^§^	**0.85 ± 0.01 [0.84; 0.87]**^§^
ACWR CP TD (AU), NST	**0.83 ± 0.03 [0.82; 0.85]**	**1.08 ± 0.05 [1.05; 1.11]**	**0.91 ± 0.03 [0.89; 0.93]**
ACWR UCP TD (AU), ST	**0.87 ± 0.02 [0.85; 0.89]**^§^	**0.99 ± 0.03 [0.94; 1.03]**^§^	**0.99 ± 0.02 [0.97; 1.02]**^§^
ACWR UCP TD (AU), NST	**0.79 ± 0.04 [0.78; 0.81]**	**1.15 ± 0.09 [1.11; 1.19]**	**1.08 ± 0.05 [1.06; 1.11]**
EWMA TD (AU), ST	**0.88 ± 0.02 [0.86; 0.89]**^§^	**0.78 ± 0.02 [0.75; 0.79]**^§^	**0.73 ± 0.02 [0.69; 0.76]**^§^
EWMA TD (AU), NST	**0.82 ± 0.03 [0.81; 0.84]**	**0.70 ± 0.04 [0.68; 0.73]**	**0.85 ± 0.07 [0.81; 0.86]**
ACWR CP HSRD (AU), ST	**0.96 ± 0.02 [0.92; 0.99]**^#^	**1.19 ± 0.06 [1.14; 1.24]**^§^	0.99 ± 0.03 [0.98; 1.02]^*^
ACWR CP HSRD (AU), NST	**0.89 ± 0.07 [0.86; 0.93]**	**1.41 ± 0.09 [1.36; 1.46]**	**0.96 ± 0.03 [0.95; 0.99]**
ACWR UCP HSRD (AU), ST	1.03 ± 0.07 [0.94; 1.12]	**1.44 ± 0.14 [1.26; 1.61]**^§^	**1.33 ± 0.09 [1.28; 1.38]**^#^
ACWR UCP HSRD (AU), NST	0.96 ± 0.18 [0.87; 1.05]	**2.06 ± 0.35 [1.89; 2.24]**	**1.20 ± 0.06 [1.15; 1.25]**
EWMA HSRD (AU), ST	1.09 ± 0.06 [0.99; 1.18]	1.36 ± 0.09 [1.25; 1.46]	1.69 ± 0.05 [1.64; 1.75]
EWMA HSRD (AU), NST	1.05 ± 0.19 [0.95; 1.14]	1.48 ± 0.21 [0.37; 1.58]	1.74 ± 0.11 [1.68; 1.79]
ACWR CP SD (AU), ST	0.97 ± 0.04 [0.94; 1.01]	1.07 ± 0.05 [1.02; 1.12]	0.87 ± 0.04 [0.84; 0.90]
ACWR CP SD (AU), NST	0.94 ± 0.07 [0.91; 0.98]	1.12 ± 0.09 [1.07; 1.17]	0.89 ± 0.05 [0.86; 0.92]
ACWR UCP SD (AU), ST	1.07 ± 0.09 [0.95; 1.18]	1.13 ± 0.07 [0.97; 1.29]	1.04 ± 0.08 [0.98; 1.09]
ACWR UCP SD (AU), NST	1.17 ± 0.23 [1.06; 1.29]	1.30 ± 0.34 [1.14; 1.47]	1.07 ± 0.09 [1.01; 1.12]
EWMA SD (AU), ST	1.13 ± 0.07 [1.04; 1.23]	1.13 ± 0.05 [1.03; 1.23]	1.13 ± 0.05 [1.06; 1.19]
EWMA SD (AU), NST	1.22 ± 0.19 [1.13; 1.32]	1.22 ± 0.21 [1.12; 1.32]	1.19 ± 0.13 [1.13; 1.27]

Significant differences between starters and non-starters are highlighted in bold (p ≤ 0.05). ACWR, acute: chronic workload ratio; EWMA, exponentially weighted moving averages; CP, coupled; UCP, uncoupled; ST, starters; NST, non-starters; s-RPE, session rate of perceived exertion; TD, total distance; HSRD, high-speed running distance; SD, speed distance.

* moderate effect,

# large effect,

§ very large effect.

The ACWR coupled of s-RPE shows a significant higher value for non-starters than starters in mid-season (*F* = 7.35; *p* = 0.014; *g* = −1.01 [−1.98, −0.09]). The ACWR coupled of TD shows a significant higher value for starters than non-starters in early-season (*F* = 47.32; *p* < 0.01; *g* = 3.01 [1.78, 4.46]) and shows a significant higher value for non-starters than starters in mid-season (*F* = 25.82; *p* < 0.01; *g* = −2.52 [−3.83, −1.39]) and end-season (*F* = 27.65; *p* < 0.01; *g* = −2.57 [−3.89, −1.43]).

The ACWR uncoupled of TD shows a significant higher value for starters than non-starters in early-season (*F* = 41.38; *p* < 0.01; *g* = 2.42 [1.31, 3.71]) and shows a significant higher value for non-starters than starters in mid-season *F* = 31.90; (*p* < 0.01; *g* = −2.28 [−3.54, −1.19]) and end-season (*F* = 25.96; *p* < 0.01; *g* = −2.26 [−3.50, −1.18]).

EWMA of TD shows a significant higher value for starters than non-starters in early-season (*F* = 22.87; *p* < 0.01; *g* = 2.25 [1.17, 3.49]) and mid-season (*F* = 21.21; *p* < 0.01; *g* = 2.42 [1.31, 3.71]) and shows a significant higher value for non-starters than starters in end-season (*F* = 25.61; *p* < 0.01; *g* = −2.23 [−3.47, −1.16]).

The ACWR coupled of HSRD shows a significant higher value for starters than non-starters in early-season (*F* = 6.52; *p* = 0.020; *g* = 1.30 [0.36, 2.33]) and end-season (*F* = 4.89; *p* = 0.040; *g* = 0.96 [0.05, 1.92]) and shows a significant higher value for non-starters than starters in mid-season (*F* = 44.58; *p* < 0.01; *g* = −2.75 [−4.14, −1.58]).

Finally, the ACWR uncoupled of HSRD shows a significant higher value for non-starters than starters in mid-season (*F* = 28.75; *p* < 0.01; *g* = −2.23 [−3.46, −1.15]) and shows a significant higher value for starters than non-starters in end-season (*F* = 13.61; *p* = 0.002; *g* = 1.63 [0.65, 2.72]).

Table 3 shows the correlation coefficient of all measures in the study for the starter’s status.

**Table 3:** Correlation analysis between external and internal load measures during three periods of the in-season for the starter’s status.

Measures	ACWR coupled s-RPE (AU)	ACWR uncoupled s-RPE (AU)	EWMA s-RPE (AU)
**Early-season**		
ACWR coupled TD (AU)	−0.256	−0.263	−0.365
ACWR uncoupled TD (AU)	−0.285	−0.305	−0.387
EWMA TD (AU)	−0.154	−0.238	−0.479
ACWR coupled HSRD (AU)	0.353	0.276	0.148
ACWR uncoupled HSRD (AU)	−0.332	−0.221	−0.010
EWMA HSRD (AU)	−0.448	−0.339	−0.182
ACWR coupled sprint (AU)	−0.338	−0.334	−0.307
ACWR uncoupled sprint (AU)	0.029	−0.029	−0.085
EWMA sprint (AU)	0.165	0.121	0.131
**Mid-season**		
ACWR coupled TD (AU)	−0.559	−0.236	−0.409
ACWR uncoupled TD (AU)	−0.448	−0.180	−0.321
EWMA TD (AU)	−0.583	−0.369	−0.577
ACWR coupled HSRD (AU)	−0.090	−0.483	0.308
ACWR uncoupled HSRD (AU)	−0.226	−0.515	0.206
EWMA HSRD (AU)	0.045	−0.394	0.574
ACWR coupled sprint (AU)	−0.338	−0.201	0.323
ACWR uncoupled sprint (AU)	−0.244	−0.134	0.381
EWMA sprint (AU)	0.467	−0.298	0.164
**End-season**		
ACWR coupled TD (AU)	−0.083	0.086	−0.176
ACWR uncoupled TD (AU)	−0.204	0.011	−0.255
EWMA TD (AU)	0.503	0.607	−0.400
ACWR coupled HSRD (AU)	0.158	0.077	0.197
ACWR uncoupled HSRD (AU)	0.222	0.160	0.079
EWMA HSRD (AU)	−0.447	−0.222	0.142
ACWR coupled sprint (AU)	0.016	−0.090	−0.541
ACWR uncoupled sprint (AU)	0.336	0.192	−0.328
EWMA sprint (AU)	**0.729**^§^	0.456	−0.150

Significant differences are highlighted in bold (*p* ≤ 0.05). ACWR, acute: chronic workload ratio; EWMA, exponentially weighted moving averages; ST, starters; NST, non-starters; s-RPE, session-rated perceived exertion; TD, total distance; HSRD, high-speed running distance.

§ very large effect, ^*^moderate effect, ^#^large effect.

Table 4 shows the correlation coefficient of all measures in the study for the non-starter’s status.

**Table 4:** Correlation analysis between external and internal load measures during three periods of the in-season for the non-starter’s status.

Measures	ACWR coupled s-RPE (AU)	ACWR uncoupled s-RPE (AU)	EWMA s-RPE (AU)
**Early-season**		
ACWR coupled TD (AU)	−0.147	−0.346	−0.463
ACWR uncoupled TD (AU)	−0.031	−0.238	−0.349
EWMA TD (AU)	0.617	0.467	0.343
ACWR coupled HSRD (AU)	0.506	0.375	0.347
ACWR uncoupled HSRD (AU)	0.149	0.028	0.042
EWMA HSRD (AU)	0.147	0.060	0.087
ACWR coupled sprint (AU)	0.208	0.260	0.291
ACWR uncoupled sprint (AU)	−0.035	0.047	0.097
EWMA sprint (AU)	0.020	0.067	0.098
**Mid-season**		
ACWR coupled TD (AU)	0.233	0.253	−0.021
ACWR uncoupled TD (AU)	0.245	0.275	−0.017
EWMA TD (AU)	0.192	0.056	−0.330
ACWR coupled HSRD (AU)	0.275	0.325	**−0.679**^#^
ACWR uncoupled HSRD (AU)	0.536	0.588	−0.592
EWMA HSRD (AU)	−0.219	−0.046	−0.398
ACWR coupled sprint (AU)	**0.801**^§^	**0.794**^§^	**−0.659**^#^
ACWR uncoupled sprint (AU)	**0.783**^§^	**0.743**^§^	−0.482
EWMA sprint (AU)	0.615	**0.640**^#^	−0.512
**End-season**		
ACWR coupled TD (AU)	−0.502	0.555	0.057
ACWR uncoupled TD (AU)	−0.408	−0.439	−0.064
EWMA TD (AU)	**−0.647**^#^	−0.561	**−0.661**^#^
ACWR coupled HSRD (AU)	−0.165	−0.252	0.245
ACWR uncoupled HSRD (AU)	−0.230	−0.302	−0.003
EWMA HSRD (AU)	0.159	0.133	−0.045
ACWR coupled sprint (AU)	0.495	0.458	0.293
ACWR uncoupled sprint (AU)	0.274	0.300	−0.041
EWMA sprint (AU)	−0.326	−0.293	−0.462

Significant differences (*p* ≤ 0.05) are highlighted in bold. ACWR, acute: chronic workload ratio; EWMA, exponentially weighted moving averages; ST, starters; NST, non-starters; s-RPE, session-rated perceived exertion; TD, total distance; HSRD, high-speed running distance.

# large effect, ^*^moderate effect,

§ very large effect.

## Discussion

The study aim was (a) to describe and compare the in-season variations of ACWR coupled, ACWR uncoupled, and EWMA through s-RPE, TD, HSRD, and sprint distance across different periods of a professional soccer season (early-season, mid-season, and end-season) between starters and non-starters; (b) to analyze the relationship the aforementioned measures across different periods of the season for starters and non-starters. The major findings from the study support our first hypothesis, given that there are some significant differences between starters and non-starters. Regarding our second hypothesis, there also were some correlations found between internal and external workload ratios for some periods, but not all workload measures showed such relationships.

Soccer is a regular and very complex system that requires constant monitoring of players’ workloads during training and especially intensive matches (Clemente et al., 2021; Silva et al., 2021). Regular monitoring of workloads allows coaches to monitor progress, training path and matches load, and to better design training based on tactical demands and team needs (Silva et al., 2021). According to Figures 1–3, most of the increases in ACWR of the parameters under consideration (s-RPE, TD, and HSRD) occurred at the beginning and end of the mid-season and at the beginning of the end-season. Also, considering the results of the season games and the existence of a draw and a loss at the beginning of the mid-season and the existence of two losses at the end of the mid-season, in order to compensate for the loss and results in the mentioned two periods, coaches have changed their game and/or training model and this has increased ACWR, consequently. These results are in line with previous studies that reported, the results of the games affect the coaches ‘expectations of the players and the game system, as well as the players’ morale, and can affect the perceived pressure, the amount of running, and other parameters (Martins et al., 2021; Silva et al., 2021).

According to Figure 4, ACWR coupled, ACWR uncoupled, and EWMA through sprint distance were higher at the beginning of the early-season than at any other time of the season. The probable reason for these findings could be less fatigue of players and their high readiness at the beginning of the season and as a result of high speeds in sprints and ball possession courses (Aquino et al., 2021).

Considering the effect size and significance level shown in Table 2, ACWR of s-RPE throughout the season shows a significantly higher value for non-starters than starters. This indicates that non-starters experienced more pressure than a certain load during the match or training. Probably due to the tightness of the matches, the use of non-starter players (as substitute players) has increased, and due to the low readiness of these players, this has caused more pressure on the non-starter players (Martins et al., 2021).

ACWR of total distance and HSRD parameters shows a significantly higher value for starters than non-starters in early-season. Also, the mentioned parameters show a significantly higher value for non-starters than starters in mid- and end-season. These results show that the non-starters have shown more workload as the season progresses. The use of non-starters in games and training has probably increased with the season’s progress to the end-season and with the fatigue or injury of starter players, which has increased the acute and chronic workload in non-starter players.

However, in the ACWR of sprint distance, no significant difference was observed between the two groups in all periods of the season (Table 2). It is also worth noting that 90 percent of professional soccer players’ sprints were under 5 s, with only 10% above 5 s. Analysis of physical loads of soccer players during matches can be useful for individualization of training of soccer players’ speed capabilities (Andrzejewski et al., 2013). It seems that since the training of the players of both groups is done together and there is no difference in the training of these players, in the ACWR of sprint distance, there is no significant difference between the players of the two groups (Aquino et al., 2021).

According to the information in Tables 3 and 4, a weak correlation and in most cases a negative correlation between external and internal load were recorded for starter players in all periods of the season (Table 3). For non-starter players (Table 4), a weak but mostly positive correlation was recorded between internal and external load in all periods of the season except mid-season. In mid-season, large and very large correlations were observed between ACWR coupled sprint and ACWR coupled s-RPE, ACWR uncoupled s-RPE, and EWMA s-RPE. Also, a very large correlation was observed between ACWR uncoupled sprint with ACWR coupled s-RPE and ACWR uncoupled s-RPE, and a large correlation was recorded in the EWMA sprint parameter with ACWR uncoupled s-RPE.

There are limitations in the present study that need to be addressed for future studies. The small number of sample size due to the limited number of players in a professional team was one of the limitations which has often been common and reported in longitudinal studies over a full season of professional competition (Nobari et al., 2020; Oliveira et al., 2021b). Also, the difference in the playing position of the players in the team has not been analyzed, while in tactics and game systems, winger positions and wide defenders have more effort and running than other players (Clemente et al., 2020; Nobari et al., 2020). In addition, the amount of sleep and the quality of nutrition of the players during the week could not be controlled, while the quality of sleep and the type of nutrition could have affected the perception of pressure, the mood of the players, and the quality of their training (Nobari et al., 2021b). Finally, a suggestion for future studies could be defining covariates to apply a repeated measures correlation which could reveal other interesting results by determine the within-individual association for paired measures assessed on two or more time points for multiple individuals (Bakdash and Marusich, 2017).

## Conclusion

As a conclusion, it seems that ACWR derived from s-RPE, total distance, high-speed running distance, and sprint distance in soccer players should be given more attention and analysis because according to the results obtained, it seems that these variables can be used to monitor the training variations and physical fitness of players. Specifically, indexes based on sprint did not allow to detect differences between playing status and the coupled version of ACWR derived from s-RPE was the only to detect differences between starters and non-starters, while the remaining variables detect differences independently or the index and variable.

As a practical application, the study’s key finding was that crucial work for non-starter players’ improvement is not done during training during the season, and these players lose preparation as the season advances. Thus, coaches should focus more on the type of training for players need to perform better when they were selected to participate in matches which will allow a better performance during the in-season. Consequently, they will have a chance to compete with the starters for a starting slot.

## Data Availability Statement

The raw data supporting the conclusions of this article will be made available by the authors, without undue reservation.

## Ethics Statement

The studies involving human participants were reviewed and approved the University of Mohaghegh Ardabili Ethics Committee prior to its start, and the Helsinki Declaration was used to follow the recommendations of Human Ethics in Research. The patients/participants provided their written informed consent to participate in this study.

## Author Contributions

HN, NA, AM, and RO: conceptualization, methodology, writing—original draft, and writing—review and editing. HN, AM, and RO: formal analysis, funding acquisition, and software. All authors have read and agreed to the published version of the manuscript.

## Funding

This research was funded by the Portuguese Foundation for Science and Technology, IP, 277 grant/award number UIDP/04748/2020.

## Conflict of Interest

The authors declare that the research was conducted in the absence of any commercial or financial relationships that could be construed as a potential conflict of interest.

## Publisher’s Note

All claims expressed in this article are solely those of the authors and do not necessarily represent those of their affiliated organizations, or those of the publisher, the editors and the reviewers. Any product that may be evaluated in this article, or claim that may be made by its manufacturer, is not guaranteed or endorsed by the publisher.
